# Health literacy, health-seeking behavior, and wellbeing among university students: insights into determinants of young adults’ health

**DOI:** 10.3389/fpubh.2026.1734575

**Published:** 2026-03-11

**Authors:** Fatma Cerit Soydan, Özlem Akman, Tülay Ortabağ, Tuba Eryigit

**Affiliations:** Department of Nursing, Faculty of Health Sciences, Istanbul Topkapı University, Istanbul, Türkiye

**Keywords:** European health literacy scale, health literacy, health-seeking behavior, PERMA, university students, wellbeing

## Abstract

**Objective:**

This study aims to investigate the associations among university students’ health literacy, health-seeking behaviors, and overall wellbeing.

**Materials and method:**

This descriptive study, grounded in a quantitative research design, was conducted among undergraduate students enrolled at a foundation university in Istanbul, Türkiye, between April and May 2024. A total of 219 students who were reached online through Google Forms and voluntarily consented to participate constituted the study sample. Data were collected using four instruments: the *Personal Information Form*, the *European Health Literacy Scale*, the *Health-Seeking Behavior Scale*, and the *PERMA Wellbeing Scale*.

**Results:**

Among the participants, 30.1% demonstrated inadequate health literacy, 38.8% had problematic health literacy, 23.7% had adequate health literacy, and 7.3% exhibited excellent health literacy. The mean score for health-seeking behavior was 2.60 ± 0.51, and the mean PERMA wellbeing score was 6.92 ± 1.53. A moderate negative correlation was identified between health literacy and health-seeking behavior, while a weak negative correlation was found between health-seeking behavior and PERMA wellbeing. Although the correlation between health literacy and wellbeing was not statistically significant, students with adequate health literacy reported higher wellbeing scores than those with problematic levels. Regression analysis revealed that higher levels of health-seeking behavior were a negative predictor of wellbeing, whereas health literacy did not significantly predict wellbeing.

**Conclusion:**

The results suggest that a substantial proportion of university students demonstrate inadequate or problematic health literacy. Although greater engagement in health-seeking behaviors may be associated with lower wellbeing, the higher wellbeing scores observed among students with adequate health literacy underscore the importance of comprehensive health education and targeted support interventions. Universities are encouraged to develop and implement programs aimed at improving health literacy, ensuring access to credible digital health resources, and reinforcing social support networks to promote overall student wellbeing.

## 1 Introduction

The World Health Organization defines health literacy as “an individual’s ability to access, understand, use, and evaluate health information and services to make informed decisions regarding the maintenance and promotion of health and wellbeing” (1). Given its critical role in shaping health behaviors and overall health status, health literacy has increasingly attracted the attention of healthcare professionals and policymakers in recent years (2). A review of the literature indicates that the Agency for Healthcare Research and Quality estimates that approximately one-third of Americans have limited health literacy, with this proportion rising to 70% among individuals aged 75 years and older (3). Similarly, a systematic review and meta-analysis assessing the prevalence of low health literacy in European Union Member States highlights that low health literacy constitutes a significant public health concern across Europe, with one in three, or nearly one in two, Europeans unable to comprehend basic health-related materials (4). A study investigating health literacy levels in Turkey reported that 30.9% of participants were classified as having inadequate health literacy, 38.0% as problematic-limited, 23.4% as adequate, and 7% as excellent (5).

Low or limited health literacy has been associated with difficulties in understanding medical information, increased healthcare costs, challenges in making informed health decisions, poorer health outcomes, and reduced participation in preventive health screening programs (6–8). University students with low health literacy have been shown to possess limited knowledge regarding sexually transmitted diseases, adhere to less nutritious diets, and face an increased risk of obesity (8). Health literacy is also a key determinant influencing individuals’ health-seeking behaviors (9).

Health-seeking behavior is defined as the set of actions undertaken by individuals to prevent illness, maintain and improve health, and seek care when needed (10–12). Consequently, health literacy and health-seeking behavior are closely interrelated. A high level of health literacy is essential for individuals to identify when and where to seek care, accurately comprehend health information, prevent illness, adhere to treatment protocols, and understand their roles in managing health-related crises (11). Studies have demonstrated that higher health literacy levels are positively associated with reduced mental health problems and enhanced subjective wellbeing (13–15). Previous studies in this field have demonstrated a significant association between health literacy and individuals’ levels of wellbeing (13–16). Evidence indicates that low health literacy is associated with poorer physical and mental wellbeing, whereas higher levels of health literacy are linked to increased subjective wellbeing (13, 17). A study conducted among adults in Turkey demonstrated that health literacy is significantly associated with general wellbeing, and that this association is further strengthened through mediating variables such as health awareness and self-awareness (16). Collectively, these findings indicate that university students’ levels of health literacy constitute a critical determinant not only of their health-related behaviors but also of their psychological and emotional wellbeing. A study conducted among the general population in Japan found that low health literacy independently predicts both poor physical and mental wellbeing (17).

However, to the best of our knowledge, no study has simultaneously examined university students’ levels of health literacy, health-seeking behavior, and wellbeing. Therefore, the present study aims to investigate the relationships among health literacy, health-seeking behavior, and wellbeing in a sample of university students.

### 1.1 Research hypotheses

*H1*: University students’ socio-demographic characteristics are associated with significant differences in their levels of health literacy, health-seeking behavior, and wellbeing.

*H2*: There is a significant relationship between university students’ health literacy levels and their health-seeking behavior and wellbeing.

*H3*: University students’ health literacy levels and health-seeking behavior significantly predict their wellbeing levels.

## 2 Materials and methods

### 2.1 Purpose and study design

This study was conducted as a descriptive, cross-sectional investigation to examine the relationships among university students’ health literacy, health-seeking behavior, and wellbeing levels.

### 2.2 Study population and sample

The study population comprised students aged 18 years and older who were enrolled at a foundation university in Istanbul. The sample consisted of 219 students who met the inclusion criteria and voluntarily agreed to participate in the study. Data were collected between April and May 2024 using Google Forms.

### 2.3 Data collection instruments

Data were collected using the *Personal Information Form*, the *European Health Literacy Scale*, the *Health-Seeking Behavior Scale*, and the *PERMA Wellbeing Scale*. Permission for the use of all scales was obtained prior to data collection.

The *Personal Information Form*: developed by the researchers based on a review of the literature (18–20), collected information on students’ age, gender, marital status, employment and income status, lifestyle, area of interest, academic department, year of study, smoking and alcohol consumption, presence of chronic diseases, overall health status, knowledge of the concept of health literacy, and participation in health literacy-related courses.

*European Health Literacy Scale:* The validity and reliability of the *European Health Literacy Scale* (21), developed by the European Health Literacy Research Consortium, were established for the Turkish population by Abacıgil et al. (22). The scale, designed to assess health literacy in individuals aged 15 years and older, comprises 47 items. The scale consists of three sub-dimensions: “Treatment Services” (items 1–16), “Prevention of Diseases” (items 17–31), and “Health Promotion” (items 32–47). Each item is rated as “very difficult” (1), “difficult” (2), “easy” (3), “very easy” (4), or “do not know” (5). Total scores range from 47 to 188, and the overall health literacy level is determined by calculating an index score using the following formula: *Index = (Arithmetic mean − 1) × (50/3).* The index score ranges from 0 (lowest health literacy) to 50 (highest health literacy). Responses marked as “do not know” (5) were excluded from the total score, index calculation, and reliability analysis, consistent with the original validity and reliability study. Based on the health literacy index calculation, health literacy levels were classified as insufficient (0–25 points), problematic–limited (26–33 points), adequate (34–42 points), and excellent (43–50 points). The internal consistency of the scale was found to be high, with a Cronbach’s alpha coefficient of 0.95.

*The Health-Seeking Behavior Scale*: Developed by Kıraç (23) to assess individuals’ health information-seeking behaviors, consists of 12 items and three subscales: Online Health-Seeking Behavior (Items 1–6), Professional Health-Seeking Behavior (Items 7–9), and Traditional Health-Seeking Behavior (Items 10–12). The scale employs a five-point Likert format, with each item scored from 1 to 5. There are no reverse-coded items. The total possible score ranges from 12 to 60, with higher scores indicating greater engagement in health-seeking behaviors. The Cronbach’s alpha coefficient of the scale was reported as 0.76 (23).

*The PERMA Wellbeing Scale:* Developed by Butler and Kern (24) to assess individuals’ levels of wellbeing, and its Turkish validity and reliability study was conducted by Demirci et al. (25). The scale consists of 15 items, 8 filler items, and 5 subdimensions, each containing 3 items. Four of the items are reverse-coded. Wellbeing is assessed based on the total of 15 items, while the filler items are evaluated separately. The scale consists of the initial letters of the following sub-dimensions. The PERMA model comprises five subdimensions:

(P) Positive Emotion: Reflects positive feelings such as happiness, joy, and comfort (Items 5, 10, and 22).

(E) Engagement: Refers to an individual’s deep involvement in a particular activity, event, or goal, experiencing a sense of flow and full absorption (Items 3, 11, and 21).

(R) Positive Relationships: Indicates satisfaction with social relationships, a sense of belonging to one’s community, and perceived social support and affection (Items 6, 15, and 19).

(M) Meaning: Represents an individual’s awareness of life purpose and the perception that life is valuable and worth living (Items 1, 9, and 17).

(A) Accomplishment: Assesses the sense of effort, achievement, and fulfillment of daily responsibilities (Items 2, 8, and 16).

In evaluating the scale, the mean score of each item within its corresponding dimension is calculated. The subdimensions do not independently determine the overall level of wellbeing; rather, they collectively contribute to it. The scale also includes eight filler items categorized under *health* (Items 4, 13, and 18), *negative emotions* (Items 7, 14, and 20), *loneliness* (Item 12), and *happiness* (Item 23). Items 7, 12, 14, and 20 are reverse-coded for negative emotions. However, since filler items are not included in the total score calculation, reverse coding is not required for scoring purposes. The Cronbach’s alpha reliability coefficient of the scale was reported as 0.91 (26).

### 2.4 Data analysis

The data obtained from the study were analyzed using the IBM Statistical Package for the Social Sciences (SPSS) version 25.0. Descriptive statistics for quantitative variables were presented as frequencies and percentages. The Kolmogorov–Smirnov test was applied to assess the normality of data distribution. For comparisons between two groups with normally distributed data, the Independent Samples *t*-test was used, whereas the Mann–Whitney *U* test was applied for data not following a normal distribution. Comparisons among three or more groups with normally distributed data were performed using one-way analysis of variance (ANOVA). The relationships between variables were tested using Pearson Correlation analysis. Multiple linear regression analysis was used to determine the effect of students’ health literacy level and health-seeking behavior on wellbeing. A *p* < 0.05 was considered statistically significant.

### 2.5 Ethical considerations

Ethical approval for the study was obtained from the Ethics Committee of Istanbul Topkapi University (Date: 07.06.2024; Decision No: 8917). Participation in the study was entirely voluntary, and informed consent was obtained from all participants prior to data collection. The English translation of the manuscript was prepared with the assistance of ChatGPT and verified by the researchers.

## 3 Findings

The mean age of the students was 24.74 ± 8.27 years. The majority were female (71.2%) and single (90.4%), while 57.5% were unemployed. Most students lived with their families (85.8%), 50.7% reported having a specific area of interest, and 63.5% were enrolled in a health-related department. Regarding lifestyle behaviors, 67.6% of students did not smoke, and 84.0% did not consume alcohol. The majority 61.6% described their general health status as good. Furthermore, 59.4% of participants reported awareness of the concept of health literacy, whereas 60.3% had not taken any health literacy-related courses (Table 1).

**Table 1:** Descriptive characteristics of students (*n* = 219).

Descriptive characteristics	*n*	%
Age mean ± SD:24,74 ± 8.27 (Min: 17–Max: 60)		
17–20	78	356
21–24	78	35.6
Age 25 and older	63	28.8
Gender		
Female	156	71.2
Male	63	28.8
Marital status		
Married	21	9.6
Single	198	90.4
Working status		
I’m working	93	42.5
I’m not working	126	57.5
Living together status		
I live with my family	188	85.8
I do not live with my family	31	14.2
Having interests such as sports and music		
Yes, I have special interests.	111	50.7
No, I do not have any special interests.	108	49.3
Department studied		
I’m studying in the health-related department.	139	63.5
I’m studying in a department outside the field of health.	80	36.5
Smoking status		
Yes, I use it.	71	32.4
No, I do not use it.	148	67.6
Alcohol use status		
Yes, I use it.	35	15.9
No, I do not use it.	184	84.0
General health status definition		
Very good	33	15.1
Good	135	61.6
Average	51	23.3
Knowledge of health literacy		
Yes, I know	130	59.4
No, I do not know.	89	40.6
Health literacy course enrollment status		
Yes, I received it.	87	39.7
No, I did not receive it.	132	60.3

The overall mean index score was 29.86 ± 8.92. For the subdimensions, the mean scores were 31.43 ± 7.98 for Treatment and Services, 29.01 ± 11.31 for Disease Prevention, and 29.09 ± 11.12 for Health Promotion (Table 2).

**Table 2:** Distribution of students’ mean index scores on the European Health Literacy Scale.

European Health Literacy Scale classification	European Health Literacy Scale 29.86 ± 8.92		Treatment and services 31.43 ± 7.98		Disease prevention 29.01 ± 11.31		Health promotion 29.09 ± 11.12	
	*n*	%	*n*	%	*n*	%	*n*	%
Insufficient health literacy	66	30.1	44	20.1	67	30.6	68	31.0
Problematic–limited health literacy	85	38.8	100	45.7	90	41.1	95	43.4
Adequate health literacy	52	23.7	59	26.9	37	16.9	35	16.0
Excellent health literacy	16	7.4	16	7.3	25	11.4	21	9.6
Total	219	100	219	100	219	100	219	100

Regarding health literacy levels, 30.1% of students were classified as having insufficient health literacy, 38.8% as problematic–limited, 23.7% as adequate, and 7.4% as excellent. The highest proportion of students with problematic–limited health literacy was observed in the Treatment and Services subdimension (45.7%), the Disease Prevention subdimension (41.1%), and the Health Promotion subdimension (43.4%). The Cronbach’s alpha coefficient of the scale was 0.94, indicating high internal consistency (Table 2).

The overall mean score for the Health-Seeking Behavior Scale was 2.60 ± 0.51. For its subdimensions, the mean scores were 2.92 ± 0.74 for Online Health-Seeking Behavior, 2.00 ± 0.65 for Professional Health-Seeking Behavior, and 2.57 ± 0.77 for Traditional Health-Seeking Behavior. The Cronbach’s alpha coefficient of the scale was 0.72. The mean total score on the PERMA Wellbeing Scale was 6.92 ± 1.53. Subdimension mean scores were 6.61 ± 2.04 for Positive Emotions, 7.26 ± 1.69 for Engagement, 6.64 ± 1.95 for Positive Relationships, 6.81 ± 1.77 for Meaning, and 7.21 ± 1.76 for Accomplishment (Table 3).

**Table 3:** Distribution of students’ mean scores on the health-seeking behavior and PERMA wellbeing scales.

Scales		Mean ± SD	Min	Max
Health-seeking behavior scale		2.60 ± 0.51	1	5
Health search behavior scale sub-dimensions	Online health search behaviour	2.92 ± 0.74	1	5
	Professional health-seeking behavior	2.00 ± 0.65	1	5
	Conventional health-seeking behavior	2.57 ±0.77	1	5
PERMA		6.92 ± 1.53	1	10
PERMA sub-dimensions	Positive emotion	6.61 ± 2.04	1	10
	Engagement	7.26 ± 1.69	1	10
	Positive relationships	6.64 ± 1.95	1	10
	Meaning	6.81 ± 1.77	1	10
	Accomplishment	7.21 ± 1.76	1	10
Filler items	Health	7.14 ± 1.81	1	10
	Negative emotions	5.75 ± 1.95	1	10
	Loneliness	5.6 ± 2.8	1	10
	Happiness	7.01 ± 2.22	1	10

For the filler items, the mean scores were 7.14 ± 1.81 for Health, 5.75 ± 1.95 for Negative Emotions, 5.60 ± 2.80 for Loneliness, and 7.01 ± 2.22 for Happiness. The Cronbach’s alpha coefficient for the PERMA scale was 0.89, indicating good internal consistency (Table 3).

A moderate, negative, and statistically significant correlation was observed between health literacy and health-seeking behavior scores (*r* = −0.330, *p* < 0.01). A weak, negative, and statistically significant correlation was found between health-seeking behavior and PERMA wellbeing scores (*r* = −0.173, *p* < 0.01). No significant correlation was detected between health literacy and PERMA wellbeing scores (Table 4).

**Table 4:** Relationship between participants’ European health literacy, health-seeking behavior, and PERMA wellbeing scores.

Assessment Scales		European health literacy	Health-seeking behavior	PERMA wellbeing scores
European health literacy	*r*	1	−0.330**	0.125
	*p*		<0.001	0.065
Health-seeking behavior	*r*	−0.330*	1	−0.173*
	*p*	<0.001		0.011
PERMA wellbeing scores	*r*	0.125	−0.173*	1
	*p*	0.065	0.011	

No significant differences were observed in mean scores based on age, gender, marital status, employment status, living arrangement, area of interest, enrollment in health-related programs, smoking or alcohol use, chronic disease status, general health status, health literacy, or health-seeking behavior. However, students who were familiar with the concept of health literacy and those who had taken courses on this subject demonstrated significantly higher health literacy scores compared to their counterparts (*p* < 0.01). These two variables, however, did not significantly affect health-seeking behavior scores (Table 5).

**Table 5:** Comparison of students’ descriptive characteristics with scale scores.

Descriptive characteristics	European health literacy			Health-seeking behavior			PERMA wellbeing scores		
	Mean ± SD	Min-Max	*p*	Mean ± SD	Min-Max	*p*	Mean ± SD	Min-Max	*p*
Age									
17–20	29.70 ± 9.37	8–49	*p* = 0.660 F = 0.146	2.62 ± 0.49	1–4	*p* = 0.069 F = 2.700	6.80 ± 1.39	3–10	*p* = 0.159 F = 1.853
21–24	30.55 ± 8.06	1.48		2.50 ± 0.51	1–4		6.75 ± 1.58	2–9	
Age 25 and older	29.20 ± 9.40	5–49		2.70 ± 0.52	2–4		7.22 ± 1.71	1–10	
Gender									
Female	29.52 ± 8.97	5–49	*p* = 0.381 *t* = 0.879	2.59 ± 0.50	1–4	*p* = 0.542 *t* = −0.610	7.00 ± 1.57	1–10	*p* = 0.165 *t* = 1.392
Male	30.69 ± 8.76	1–49		2.64 ± 0.53	1–4		6.67 ± 1.53	3–10	
Marital status									
Married	30.18 ± 8.60	5–48	*p* = 0.104 *t* = 1.634	2.77 ± 0.51	1–4	*p* = 0.128 *t* = −1.530	7.69 ± 1.21	5–10	*p* = 0.015^*^ t = −2.462
Single	26.85 ± 11.21	1–49		2.59 ± 0.51	1–4		6.82 ± 1.57	1–10	
Working status									
I’m working	29.84 ± 9.17	1–48	*p* = 0.974 *t* = −0.033	2.59 ± 0.56	1–4	*p* = 0.730 *t* = −0.345	7.00 ± 1.63	1–9	*p* = 0.143 *t* = 0.821
I’m not working	29.88 ± 8.75	6–49		2.61 ± 0.47	1–4		6.83 ± 1.51	2–10	
Living together status									
I live with my family	29.43 ± 8.82	5–49	*p* = 0.079 *t* = −1.764	2.62 ± 0.50	1–4	*p* = 0.139 *t* = 1.484	6.99 ± 1.49	2–10	*p* = 0.045^*^ t = 2.019
I do not live with my family	32.46 ± 9.18	1–49		2.48 ± 0.56	1–4		6.38 ± 1.86	1–9	
Having interests such as sports and music									
Yes, I have special interests.	30.52 ± 7.75	11–49	*p* = 0.268 *t* = 1.111	2.60 ± 0.52	1–4	*p* = 0.830 *t* = −0.215	7.23 ± 1.43	2–10	*p* = 0.002^*^ t = 3.125
No, I do not have any special interests.	29.18 ± 9.96	1–49		2.61 ± 0.50	1–4		6.59 ± 1.63	1–10	
Department studied									
I’m studying in the health-related department.	30.26 ± 8.92	1–49	*p* = 0.387 *t* = 0.867	2.57 ± 0.51	1–4	*p* = 0.194 *t* = 0.897	6.97 ± 1.66	1–10	*p* = 0.378 *t* = 0.883
I’m studying in a department outside the field of health.	29.17 ± 8.91	5–49		2.66 ± 0.51	1–4		6.78 ± 1.37	3–10	
Smoking status									
Yes, I use it.	30.14 ± 9.30	8–49	*p* = 0.747 *t* = 0.323	2.63 ± 0.55	1–4	*p* = 0.618 *t* = 0.500	6.79 ± 1.78	1–10	*p* = 0.453 t = −0.752
No, I do not use it.	29.72 ± 8.75	1–48		2.59 ± 0.49	1–4		6.96 ± 1.45	3–10	
Alcohol use status									
Yes, I use it.	28.95 ± 8.04	12–49	*p* = 0.520 *t* = −0.645	2.68 ± 0.66	1–4	*p* = 0.452 *t* = 0.760	6.41 ± 1.72	1–9	*p* = 0.046^*^ t = −2.006
No, I do not use it.	30.03 ± 9.07	1–49		2.59 ± 0.48	1–4		6.99 ± 1.52	2–10	
General health status definition									
Very good	31.67 ± 8.46	12–49	*p* = 0.123 F = 3.034	2.49 ± 0.60	1–4	*p* = 0.256 F = 2.877	7.21 ± 1.80	3–10	*p* = 0.004^*^ F = 5.603
Good	30.19 ± 8.12	6–49		2.60 ± 0.50	1–4		7.06 ± 1.35	3–10	
Average	27.83 ± 10.81	1–45		2.68 ± 0.48	1–4		6.28 ± 1.77	1–9	
Knowledge of health literacy									
Yes, I know	31.40 ± 8.77	1–49	*p* = 0.002^*^ t = 3.156	2.55 ± 0.54	1–4	*p* = 0.055 *t* = −1.931	7.08 ± 1.58	1–10	*p* = 0.047^*^ t = 2.002
No, I do not know.	27.61 ± 8.68	5–49		2.68 ± 0.46	1–4		6.65 ± 1.51	3–10	
Health literacy course enrollment status									
Yes, I received it.	31.67 ± 9.13	1–49	*p* = 0.014^*^ t = 2.474	2.56 ± 0.51	1–4	*p* = 0.272 *t* = −1.100	7.14 ± 1.69	1–10	*p* = 0.064 *t* = 1.861
No, I did not receive it.	28.66 ± 8.59	5–49		2.63 ± 0.51	1–4		6.74 ± 1.45	3–10	

*F* = ANOVA test *t* = Student *t*-test *p < 0.05.

Regarding PERMA wellbeing scores, students who were married (*p* < 0.05), lived with their families (*p* < 0.05), had a specific area of interest (*p* < 0.01), abstained from alcohol (*p* < 0.05), reported their general health as very good (*p* < 0.01), and were familiar with the concept of health literacy (*p* < 0.05) exhibited significantly higher wellbeing scores (Table 5).

The results indicated that students with adequate health literacy had significantly higher PERMA scores compared to those with problematic–limited health literacy (*p* < 0.05) (Table 6).

**Table 6:** Comparison of PERMA wellbeing scale mean scores between students with problematic–limited and adequate health literacy levels.

Health literacy level	*n*	%	Mean ± SD	PERMA total score		
				*t*-test		
				*t*	SD	*p*
Problematic–limited health literacy	152	69.0	6.77 ± 1.53	−2.16	217	0.031*
Adequate health literacy	67	31.0	7.25 ± 1048			

*p < 0.05.

The analysis aimed to determine the extent to which health literacy and health-seeking behavior predict overall wellbeing. Prior to conducting the regression analysis, the assumptions of normality, linearity, and autocorrelation were evaluated. Descriptive statistics indicated that skewness and kurtosis values for all variables were within the ±1 range (Health Literacy = −0.064, PERMA = −0.492, Health-Seeking Behavior = −0.394; kurtosis values = 0.535, 0.367, and 0.413, respectively), suggesting that the normality assumption was met. Autocorrelation was assessed using the Durbin-Watson test, yielding a coefficient of 1.21. Furthermore, multicollinearity diagnostics indicated no multicollinearity issues between the dependent variable (wellbeing) and the independent variables (health literacy and health-seeking behavior) (Table 7).

**Table 7:** Multiple linear regression analysis of health literacy and health-seeking behavior scores as predictors of PERMA wellbeing scale total scores.

Dependent variable: wellbeing							
Independent variable: health literacy, health-seeking behavior	*B*	Standard error	*β*	*t*	*p*	Tolerans	VIF
Fixed	7.454	0.916		8.142	<0.001		
Health literacy	0.005	0.004	0.076	1.078	0.282	0.891	1.122
Health-seeking behavior	−0.037	0.018	−0.147	−2.082	0.039	0.891	1.122
R: 0.187, *R*^2^: 0.035, Revised *R*^2^: 0.026, Durbin-Watson: 1.21, Model için *F*_2−216:_ 3.915; *p* < 0.05							

The explanatory power of the model developed in this study was low but statistically significant, with the independent variables collectively accounting for approximately 3.5% of the variance in students’ wellbeing levels (*R*^2^ = 0.035). These findings suggest that health literacy and health-seeking behavior may have a limited, yet meaningful, role in predicting overall wellbeing. Examination of the individual predictors revealed that students’ health-seeking behavior scores significantly and negatively predicted their psychological PERMA wellbeing (*β* = −0.147, *t* = −2.082, *p* < 0.05), indicating that higher engagement in health-seeking behaviors may be associated with lower wellbeing levels. In contrast, although health literacy scores showed a positive association with psychological wellbeing, this effect was not statistically significant (*β* = 0.076, *t* = 1.078, *p* > 0.05) (Table 7).

## 4 Discussion

The present study aimed to assess university students’ health literacy, health-seeking behavior, and PERMA wellbeing levels, and to examine the relationships among these variables.

The findings of the present study indicate that the majority of university students (69%) exhibit inadequate or problematic levels of health literacy. Furthermore, the overall mean health literacy score was classified within the problematic range. This result is consistent with findings reported in several previous studies conducted among university student populations.

Previous studies have similarly reported high proportions of university students with inadequate or problematic levels of health literacy. For instance, Ergün (27) found that 59.9% of university students demonstrated inadequate or limited–problematic health literacy skills. Likewise, Rosario et al. (28) reported that 82.3% of students exhibited limited health literacy, encompassing both inadequate and problematic levels, while Evans et al. (29) observed that 55% of university students fell within the limited health literacy category. Similarly, Akgün et al. (30) reported low mean health literacy scores among nursing students. In contrast, Rueda-Medina et al. (31), in a study conducted with health sciences students in Spain, found that the majority of participants (69.5%) demonstrated adequate or excellent levels of health literacy, with a mean score of 36.52 ± 7.73. Similarly, the study conducted by Güllü et al. (32) among health sciences students indicated that students generally exhibited adequate levels of health literacy. Nevertheless, evidence from the literature suggests that, irrespective of the predominant literacy level reported, a considerable proportion of university students continue to demonstrate inadequate or problematic health literacy. These findings underscore the need for targeted interventions aimed at improving health literacy levels within university populations.

The literature also includes studies conducted across different age groups. Abacıgil et al. (22) reported that 52.7% of individuals aged between 17 and 87 years exhibited inadequate or problematic levels of health literacy. Similarly, Bakan and Yıldız (33) found that 77.8% of individuals aged 21–64 years had inadequate or limited health literacy. Collectively, these findings indicate that inadequate and limited health literacy constitutes an important public health concern not only among university students but also within the broader population.

In the present study, the examination of health literacy levels according to student characteristics revealed that higher health literacy scores were observed only among students who were familiar with the concept of health literacy and who had received education or coursework on this subject. Consistent with these findings, previous studies have reported that individuals who participate in health-related courses demonstrate higher levels of health literacy (28, 33). The association between awareness of health literacy and formal education in this area and higher health literacy levels underscores the critical role of educational interventions in improving health literacy.

However, contrary to the findings of the present study, several studies have reported higher health literacy levels among female students (27, 28, 32), male students, individuals who perceived their health status as better (34), those who were satisfied with their health status, and those who reported having sufficient financial resources (28). In contrast, other studies, in line with the results of the present study, found that variables such as living with family (34), age, gender, employment status, and the presence of chronic disease did not significantly influence health literacy levels (17). Collectively, these findings suggest that health literacy levels may vary according to the socio-demographic and contextual characteristics of the study populations.

The findings of the present study indicate that students most frequently engage in online health-seeking behavior, whereas professional health-seeking behavior was observed at the lowest level. This pattern suggests that students predominantly rely on the internet as their primary source of health-related information. Consistent with this finding, Basch et al. (35) reported that 74% of university students use the internet to obtain health information. In contrast, other studies have documented higher levels of professional health-seeking behavior and lower levels of online health-seeking behavior (9, 36). Nevertheless, the predominance of online health-seeking behavior among students in the present study underscores the critical importance of safe and accurate internet use, particularly in relation to digital health literacy.

The results of the present study indicate that the characteristics of the participating students did not result in significant differences in their health-seeking behavior. Consistent with these findings, Deniz and Çimen (9) reported that health-seeking behaviors do not vary according to participants’ gender, age, education level, or employment status. Similarly, Arı (36) found that variables such as age, gender, living arrangements, family income, the presence of chronic diseases in individuals or their families, and employment status were not significant determinants of health-seeking behavior. Collectively, these findings suggest that socio-demographic characteristics may not be primary determinants of health-seeking behavior.

In the present study, the PERMA wellbeing scores of participating students ranged from 6.61 to 7.26, with a mean score of 6.92 ± 1.53. Comparable findings have been reported in previous research. For instance, Sun et al. (37) observed PERMA wellbeing scores ranging from 6.27 to 7.10 among nursing students in China. Similarly, a study conducted in Turkey reported a mean PERMA wellbeing score of 6.78 ± 1.23, which is consistent with the results of the present study (25). Furthermore, Günaydın and İnal (38), in a study involving 1,101 participants, found that individuals aged 18–25 years—who constituted 43.9% of their sample—had lower psychological wellbeing (PERMA score: 6.88 ± 1.25) compared to other age groups included in the study. Notably, 71.2% of the students in the present study also fell within this age range. Overall, the similarity of PERMA wellbeing scores across these studies highlights the importance of implementing initiatives aimed at enhancing wellbeing, particularly among university student populations.

An examination of the PERMA subscales in the present study revealed that students scored highest on the engagement (attachment) dimension, whereas the positive emotions dimension yielded the lowest scores. In a study conducted by Demirci et al. (25) with 253 university students, life satisfaction was reported as the highest dimension, while positive emotions and engagement were the lowest. In contrast, Sun et al. (37) found that the relationships dimension had the highest scores, whereas the meaning and accomplishment dimensions were the lowest. A comparison of these findings indicates that the relative ranking of PERMA subdimensions varies across studies. Nevertheless, it is noteworthy that the positive emotions dimension was consistently reported as relatively low in the present study and in other studies conducted in Turkey. This pattern suggests that initiatives aimed at enhancing wellbeing should take this dimension into particular consideration and place greater emphasis on promoting positive emotions.

In the present study, higher levels of wellbeing were observed among students who were married, lived with their families, had personal interests, did not consume alcohol, perceived their general health status as very good, and were familiar with the concept of health literacy. Notably, the positive differentiation of wellbeing according to factors that may be conceptualized as indicators of social support—such as living with family, being married, and having an area of personal interest—is consistent with the findings reported by Boyacı (39) and Liu et al. (40). Boyacı (39) demonstrated a positive association between university students’ wellbeing and social support scores, further indicating that positivity and social support significantly predicted PERMA-based wellbeing. Similarly, Liu et al. (40) identified a positive relationship between social support, subjective wellbeing, and health literacy, and reported that social support played a moderating role in the relationship between health literacy and wellbeing. Collectively, these findings support the results of the present study and underscore the critical importance of both social support and health literacy in promoting wellbeing.

The findings of the present study revealed a negative association between students’ health literacy levels and their health-seeking behaviors. A review of the literature indicates that these results differ from those reported in previous studies. For instance, Mansur and Ülke (41), in a study conducted with university students, identified a positive relationship between health literacy and health-seeking behavior. Similarly, Şantaş et al. (11) reported a weak positive association between health literacy and health-seeking behavior among individuals attending a family health center. The negative relationship observed in the present study may be interpreted as suggesting that individuals with higher levels of health literacy experience fewer health-related uncertainties or concerns and, consequently, may exhibit a reduced need to engage in health-seeking behaviors.

The findings of the present study did not reveal a significant direct relationship between students’ health literacy levels and their PERMA-based wellbeing. Nevertheless, students with problematic or inadequate health literacy were found to have lower levels of wellbeing compared to those with adequate or excellent health literacy. Previous research has demonstrated associations between health literacy and wellbeing, healthy lifestyle behaviors (2, 30, 40), mental wellbeing (8), and both physical and mental health outcomes (17). Furthermore, Rueda-Medina et al. (31) reported that health literacy is associated with health-promoting lifestyle behaviors and physical activity, and that limited health literacy (inadequate or problematic) is linked to unhealthy behaviors. Collectively, these findings may help explain the lower wellbeing observed among students with problematic or inadequate health literacy in the present study. It can be suggested that students with limited health literacy may experience difficulties in accessing, interpreting, and integrating health-related information into their daily lives, which may, in turn, be associated with reduced wellbeing.

The present study demonstrated that higher levels of health-seeking behavior were associated with lower PERMA-based wellbeing among students. In line with this finding, Yaman and Atalay (42) reported that increases in self-awareness and health perception particularly regarding the perceived importance of health were accompanied by decreases in health-seeking behavior. The negative association observed between health-seeking behavior and PERMA wellbeing in the present study may be interpreted as indicating that individuals with higher levels of wellbeing experience fewer health-related concerns and, therefore, engage less frequently in health-seeking behaviors. Conversely, individuals with lower PERMA wellbeing may be more inclined to engage in health-seeking behaviors, as such behaviors generally reflect efforts to address perceived or experienced health-related problems.

The present study examined the effects of participants’ health literacy and health-seeking behaviors on their PERMA-based wellbeing. The findings indicated that both health literacy and health-seeking behavior were significant predictors of wellbeing; however, the overall explanatory power of the model was relatively low. This suggests that wellbeing is influenced by multiple factors and cannot be explained solely by health literacy and health-seeking behavior. Furthermore, health literacy levels were not found to have a direct effect on students’ PERMA wellbeing. In contrast, increased health-seeking behavior particularly health-related searching was associated with lower PERMA wellbeing. In this context, Starcevic and Berle (43) reported that individuals experiencing health-related concerns and anxiety often engage in repetitive and excessive health searches, especially via the internet, which may exacerbate fear and uncertainty rather than provide reassurance. These findings may help explain the negative association observed between health-seeking behavior and wellbeing in the present study. Similarly, Muse et al. (44) reported that individuals with elevated levels of health anxiety tend to engage more frequently in online health-seeking behavior, and that such behavior is associated with increased distress and anxiety. These findings may help explain the negative association between health-seeking behavior and wellbeing observed in the present study. It can be suggested that as students’ engagement in health-related search behaviors increases, their levels of stress and anxiety may also rise, which may, in turn, be associated with lower levels of wellbeing.

### 4.1 Strengths and limitations of the study

The findings of this study are limited to volunteer students who were enrolled at the specified foundation university in Istanbul during a defined time period and who met the inclusion criteria. Therefore, the results cannot be generalized to the wider university student population. Despite this limitation, the relatively limited number of studies focusing on university students in this field constitutes a notable strength of the present study, as it contributes to the existing literature. Moreover, the simultaneous examination of health literacy, health-seeking behavior, and PERMA-based wellbeing variables adds further depth and originality to the study.

## 5 Conclusion

University education represents a developmental period during which students increasingly assume responsibility for themselves and make autonomous decisions. During this stage, health-related behaviors are shaped predominantly by students’ personal decisions and lifestyle choices rather than by the influence of family members or other adults. Consequently, the choices made during this period are likely to have lasting implications for individuals’ health status and overall wellbeing in adulthood.

The findings of this study indicate that university students generally exhibit inadequate or problematic levels of health literacy and predominantly engage in online health-seeking behavior. Students with inadequate and problematic health literacy were found to have lower levels of wellbeing. Moreover, a negative association was observed between health-seeking behavior and PERMA-based wellbeing. Although health literacy and health-seeking behavior emerged as significant predictors of wellbeing, the overall explanatory power of the model was relatively low. These results suggest that increased engagement in health-seeking behavior, particularly in online contexts, may be associated with lower levels of wellbeing.

Our study reveals that the majority of university students have inadequate and problematic health literacy, highlighting the need for research in this area. It is recommended that health literacy education be integrated into the curriculum to improve wellbeing. In particular, given that our study found higher levels of online health search behavior among students, awareness training on digital health literacy could be provided to support students in critically evaluating online health information. Such initiatives are considered to have the potential to support wellbeing by reducing anxiety-based health search behavior.

In particular, the relationship between wellbeing and social support may indicate that social support systems (peer counseling, psychological counseling centers, etc.) can be used to increase wellbeing. The fact that students with a particular area of interest have higher levels of wellbeing highlights the importance of increasing and encouraging social activities at universities in this regard.

## Data Availability

The original contributions presented in the study are included in the article/supplementary material, further inquiries can be directed to the corresponding author.
